# Inhibitory evaluation of *Curculigo latifolia* on α-glucosidase, DPP (IV) and *in vitro* studies in antidiabetic with molecular docking relevance to type 2 diabetes mellitus

**DOI:** 10.1080/14756366.2020.1844680

**Published:** 2020-11-29

**Authors:** Nur Athirah Zabidi, Nur Akmal Ishak, Muhajir Hamid, Siti Efliza Ashari, Muhammad Alif Mohammad Latif

**Affiliations:** a Laboratory of Molecular Biomedicine, Institute of Bioscience, Universiti Putra Malaysia, Serdang, Selangor, Malaysia; b Centre of Foundation Studies for Agricultural Science, Universiti Putra Malaysia, Serdang, Selangor, Malaysia; c Department of Microbiology, Faculty of Biotechnology and Molecular Science, Universiti Putra Malaysia, Serdang, Selangor, Malaysia; d Integrated Chemical Biophysics Research, Faculty of Science, Universiti Putra Malaysia, Serdang, Selangor, Malaysia

**Keywords:** α-glucosidase, DPP (IV), *Curculigo latifolia*, molecular docking, type 2 diabetes

## Abstract

The inhibition of α-glucosidase and DPP enzymes capable of effectively reducing blood glucose level in the management of type 2 diabetes. The purpose of the present study is to evaluate the inhibitory potential of α-glucosidase and DPP (IV) activity including with the *2-*NBDG uptake assay and insulin secretion activities through *in vitro* studies. The selected of active compounds obtained from the screening of compounds by LC-MS were docked with the targeted enzyme that involved in the mechanism of T2DM. From the results, root extracts displayed a better promising outcome in α-glucosidase (IC_50_ 2.72 ± 0.32) as compared with the fruit extracts (IC_50_ 3.87 ± 0.32). Besides, root extracts also displayed a better activity in the inhibition of DPP (IV), enhance insulin secretion and glucose uptake activity. Molecular docking results revealing that phlorizin binds strongly with α-glucosidase, DPP (IV) and Insulin receptor (IR) enzymes with achieving the lowest binding energy value. The present work suggests several of the compounds have the potential that contribute towards inhibiting α-glucosidase and DPP (IV) and thus effective in lowering post-prandial hyperglycaemia.

## Introduction

Diabetes mellitus (DM) is a chronic carbohydrate metabolism disorder that has an abnormal homeostasis of glucose leading to a high blood glucose levels.Globally, diabetes mellitus (DM) is recognised as one of the major disease in the world with the highest crisis diseases in the 21st century^1^. According to the International Diabetes Federation in the 9^th^ edition, it has been revealed that approximately 425 million adults between ages 20–79 years were diagnosed with diabetes worldwide in 2019. However, if this does not recover or reduced, this figure is expected to rise around 700 million by 2045 and based on records, almost 49.7% was undiagnosed and living without knowing they had this disease^2^. Globally, based on the records, 374 million of people is at risk due to the developing in type 2 diabetes mellitus (T2DM), hence these evolution is increasing awareness of people about T2DM^3^.

Type 2 diabetes mellitus or shortly known as T2DM is a type of diabetes disease that has features of insulin resistance from the decrease in the peripheral glucose uptake in most of the targeted tissue. This is happen due to the insensitivity of insulin from the insulin resistance that may reduce the production of insulin and finally leading to excessive blood glucose level or known as hyperglycaemia^4^. Recently, the pathogenies of T2DM is intertwined with various type of mechanism such as increasing insulin secretion, insulin sensitivity, increased response towards incretin hormones, decrease reabsorption of glucose through carbohydrate digestion and increased in peripheral glucose uptake^5^. One of the most potent approaches in the management of T2DM is by Dipeptidyl peptidase (IV) inhibition, where the inhibitor of these enzymes will prevent the degradation of the incretin hormone, which in turn increase the insulin secretion and lowering blood glucose level^6^. Apart from this, the excessive amount of carbohydrates present in the diet can be considered as one of the major causes of diabetes. The delaying absorption of sugar in the bloodstream that may exert in low blood sugar level which through an inhibitory potential of α-glucosidase, known as the main enzyme in the carbohydrate mechanism. The inhibition of these enzymes is able to delay the absorption of monosaccharides from dietary complex carbohydrates, preventing any sudden rise in meal-induced blood glucose level, and therefore are involved in controlling the glucose level in the blood^7^. This is well correlated with the interesting finding that reveals that the α-glucosidase enzyme inhibitor is capable of slowing carbohydrate digestion, resulting in less efficient glucose absorption and thus decreasing postprandial hyperglycaemia^8^.

Treatment in T2DM is mostly by consuming the oral antidiabetic drugs that act through the different mechanisms in controlling the increased blood glucose level along with changes in diet and healthy lifestyles. Clinically, there is various type of oral hyperglycaemic drugs, which has been used in controlling this disease and are classified into different classes such as biguanides, sulfonylureas, thiazolidinediones (TZD), meglitinides, Dipeptidyl peptidase (IV) inhibitors, sodium-glucose cotransporter (SGLT2), and α-glucosidase inhibitors^9^. Each of the classes is targeting a different type of organ and are carried out in the various mode of action. In fact, usually, combinations of different antidiabetic drugs are used to increase the efficacy of the treatment^10^. However, despite its benefits, these drugs also have their own long-term effects that can give out adverse effects caused by chronic administration, including cardiovascular disease, lactate acidosis, hypoglycaemia, gastrointestinal complaints and others^11^. To date, molecular docking studies have been used recently in predicting the potential inhibitor that has been compared with marketed anti-diabetic drugs as which by seeking these natural compounds as new anti-diabetic agents with little or no adverse effects for the long term effect. Molecular docking is an important computational method for predicting possible drug-protein interactions with the selected ligand that represent bioactive compound containing in the medicinal plants^12^.

Nowadays, a new approach is introduced by applying plant extracts, which may contain high therapeutic values as an alternative way of mimicking the function of antidiabetic drugs and also have been reported to contain anti-diabetic properties^13^. Likewise, *Curculigo latifolia* Dryand. ex W.T.Aiton, *or* locally known as lemba is a rhizome geophyte that is categorised under family Hypoxidaceae. From the previous study, *C. latifolia* has been revealed has the ability to exhibit antidiabetic properties through cell lines and in diabetic induced rats^14^. The root extract of *C. latifolia* has shown to modulate glucose and lipid metabolism in experimental diabetic rats and the presence of curculigoside may have bestowed its anti-diabetic efficacy^14^^,^^15^. Nonetheless, there were no previous studies have yet been revealed on the inhibition of DPP (IV), α-glucosidase assays, and molecular docking and this present research could be regarded as an additional confirmation of antidiabetic properties for this crop. The *C latifolia* root extracts were previously screened for total phenolic content (TPC), total flavonoid content (TFC), antioxidant activity and screening of active compounds through LCMS analysis^16^. Therefore, the current studies are conducted with an objective to evaluate antidiabetic properties in the *C. latifolia* root and fruits extracts with the use of environmentally extraction’s technique, which is subcritical water extraction (SWE) method through inhibitory activities against α-glucosidase and DPP (IV) enzyme along with *in vitro* studies by examining the glucose uptake and insulin secretion. Additionally, molecular docking simulation was also been performed in order to predict the molecular docking scores by identifying the docking score in each targeted protein involved.

## Materials and methods

### Plant materials

The *C. latifolia* plant was collected in Selangor, Malaysia. (Geographical Coordinates: 2.8833° N, 101.8667° E). A voucher specimen of the plant (SK 1709/09) was confirmed and deposited in the Biodiversity Unit, Institute of Bioscience, Universiti Putra Malaysia, Serdang, Selangor, Malaysia. The fruits are plucked and cleaned with the tap water blotted with tissue paper and stored at −20 °C for overnight and freeze-dried. Roots are cleaned with tap water and subsequently dried overnight in an oven at 40 °C. The dried roots and fruits were ground into a fine powder that passed through a 30-mesh sieve. The powdered roots and fruits were sealed and kept at 4 °C for future use.

### Materials and reagents

Methanol, acetonitrile, formic acid and ethanol (HPLC grade) were purchased from Merck (Darmstadt, Germany). All other chemicals were of analytical grade purity. All aqueous solutions were prepared using ultrapure water. Drugs (acarbose, sitagliptin), enzymes alpha-glucosidase from *Saccharomyces cerevisiae*, substrates (4-nitrophenyl β-D-glucopyranoside) and were purchased from Sigma Chemical Co. (USA). The DPP (IV) inhibitor screening assay kit was obtained from Cayman Chemicals (Catalog no. 700210) and was stored at −80 °C.

### Preparation of extract through SWE method

Subcritical water extraction (SWE) was carried out using a batch system comprising of a SEPAREX piston pump (P200 LGP50, France), an electrical control panel and an extraction vessel. The total volume of the high-pressure stainless steel vessel was 50 ml, thus, it was filled with about 2 g of the *C. latifolia* root and fruit extract respectively. Similar to what was done in our previous research work, the independent variables were measured at optimum conditions which were set at 180 °C for 30 min of extraction time^16^. All other parameters were held constant, comprising of an extraction pressure of 100 bar, the flow rate of 0.5 ml/min, and water to the solvent ratio of 1:10 *w/v*. After extraction, the extraction vessel was cooled and depressurised. The obtained extracts were then concentrated by drying in a freeze dryer to remove any excess water completely. The pure extract was then stored in a dark place at 4 °C for further analysis.

### Measurement of enzymatic inhibitory activity

#### α-Glucosidase inhibitory assay

The α-glucosidase inhibition activity of the sample was determined following the procedure described with minor modifications^17^. A 40-μL aliquot of α-glucosidase solution (1 U/mL) is added to 1μLof sample solution (500 μg/mL in DMSO) and 69 μL of 0.1 M sodium phosphate buffer. After 15 min incubation at 37 °C, 40 μL of substrate solution (5 mM p-nitrophenyl α-D- glucopyranoside) is added to the reaction mixture and incubated at 37 °C for 30 min. Then, the reaction is terminated by adding 150 μL of 0.1 M Na_2_CO_3_. The yellow product can be determined at absorbance 405 nm, using a plate reader (Biotek Synergy H1 Multi-Mode Reader, Winooski, VT, USA). The percentage of inhibition is calculated as follows:

$$\text{\% inhibition}=\;(1-A_{\mathrm{sample}}/A_{\mathrm{control}}\;)\text{ × }100$$

Reference acarbose also tested as a positive control in this assay. All samples were analysed in triplicate.

#### DPP (IV) inhibitory assay

The inhibition of DPP (IV) was conducted by following DPP (IV) screening assay kit by Cayman Chemical (Michigan, USA), (Item no: 700210). The positive control inhibitor, 30 µl of sitagliptin and sample was made in varying concentrations (500–8000 µg/ml) in an assay buffer was added into10 µl of DPP (IV) enzyme solution. Then, the solutions were incubated for about 15 min at 37 °C. A 50 µl of the substrate was added into the mixture and was incubated for 30 min at 37 °C. The fluorescence reading was measured by setting excitation wavelength at 355 nm, emission wavelength at 455 nm and gain setting at 80. The percentage of inhibition was calculated as follows:

$$\text{\% inhibition}=\;(1-A_{\mathrm{sample}}\;/A_{100\text{\% initial activity}})\times 100$$

Where 100% initial activity is formed by the combination of assay buffer, DPP (IV) and distilled water. Sitagliptin is used as a positive control in this assay. All samples are analysed in triplicate.

### Liquid chromatography-mass spectrometry (LC-MS) analysis

LC-MS analysis was performed using MS-Q-TOF mass spectrometer (Agilent Technologies, Santa Clara, USA) coupled to an HPLC system (Agilent Technologies, Santa Clara, USA). A C18 column (125 × 2 mm i.d., 5 μm particle size) was used. The mobile phase was composed of water (A, 6% acetic acid) in acetonitrile (B, 1% acetic acid), the gradient is as follows: 2–5.00 min; 100% A, 5–10 min 60% A, 10–15 min 50% A, 15–20 min 20%. The sample injection volume was 10 μl and the mobile phase flow rate was 0.5 ml/min. The column of chromatography was kept at 30 °C. Based on the available mass spectra found in the literature libraries, the identified recognised active compounds are significantly identified.

### In vitro studies

#### Cell culture

All the cells including 3T3-L1 preadipocyte cell, L6 muscle cell, and BRIN BD11 pancreatic cells were obtained from Dr. Muhajir Hamid, Animal Cell Culture Laboratory, Faculty of Biotechnology and Biomolecular Sciences, Universiti Putra Malaysia. BRIN BD11 cells were cultured in RPMI growth medium supplemented with 10% (*v/v*) foetal calf serum (FBS, Gibco/BRL), 100 µg mL − 1 penicillin and 10 µg mL − 1 streptomycin (Gibco/BRL) at 37 °C in 5% CO_2_. Cells were passaged every 5–7 days. L6 muscle cell and 3T3 adipose cell is maintained in DMEM supplemented with 10% inactivated Foetal Bovine Serum (FBS), penicillin (100 IU/ml) and streptomycin (100 g/ml) in a humidified atmosphere of 5% CO_2_ at 37 °C until confluent. The passage number of cell used was between 20 to 30.

#### Cell cytotoxicity by MTT assay

Cell viability was assessed by MTT colorimetric assay as reported with minor modifications^18^. Cells were seeded at a density of 1 × 10^5^ cells/well in a 96-well plate and allowed to attach for 24. The attached cells were then exposed to the test samples with varying concentrations for a 24 h challenge. Subsequently, 20 μL of MTT (5 mg/mL) was added and further incubated for another 4 h period. Following removal of excess MTT and PBS rinse, 200 μL of DMSO was added to dissolve the formazan crystals formed. The purple formazan coloured product was quantified at 570 nm, using a plate reader (Biotek Synergy H1 Multi-Mode Reader, Winooski, VT, USA). In this assay, 1% of DMSO was employed as vehicle control and Triton X-100 (1.0%; *v/v*) in DMEM were being employed as a positive control. The cell viability was expressed as a fraction of viable cells relative to vehicle control cultures. The IC_20_ values and IC_50_ values, indicating the respective concentration of test sample producing 20 and 50% inhibition in growth, were calculated using Graphpad Prism 7.0 (San Diego, California, USA).

#### 3T3–L1 pre-adipocyte differentiation

Differentiation of 3T3-L1 cells was performed following the guideline provided by American Type Culture Collection (ATCC) Technical Bulletin No. 9 (ATCC, 2011). The cells were first seeded into 24-well culture plate at a density of 3 × 10^4^ cells/well and further incubated until 100% confluence. The differentiation process (Day 1) was initiated by incubating the cells in differentiation medium comprising of DMEM with 10% FBS, 50 μg/mL gentamicin, 1.0 µM dexamethasone, 0.5 mM IBMX and 1.0 µg/mL insulin. Fresh differentiation medium was supplied after every 48 h of incubation. On day 5, the differentiation medium was replaced with an adipocyte maintenance medium consisting of DMEM with 10% FBS and 1.0 µg/mL insulin. The replacement of fresh medium after every 48 h incubation period proceeded until day 12. Control cultures were treated with either 0.1% DMSO (20 µL) or rosiglitazone (5–20 µM).

#### 2-Nbdg uptake assay

Stimulated glucose uptake activity was performed using fluorescently-labelled 2 NBDG glucose analog based on modified protocol^19^. The protocol was followed based on the 2-NBDG uptake cell-based assay kit (Item No: 600470) Cayman Chemical (Michigan, USA). Glucose uptake activity of test drugs is determined in L6 and 3T3-L1 cells on 96-well clear bottom black fluorescence plates (Thermo Scientific, Pittsburgh, PA, USA). In brief, the 24 h cell cultures with 70–80% confluency are allowed to differentiate by maintaining in DMEM with 2% FBS for 4–6 days. The extent of differentiation is established by observing the multinucleation of cells. The differentiated cells are serum-starved overnight and at the time of the experiment, cells are washed with HEPES buffered Krebs Ringer Phosphate solution (KRP buffer) once and incubated with KRP buffer with 0.1% BSA for 30 min at 37 °C. Cells are treated with different non-toxic concentrations of test sample (1.5–0.0468 mg/mL) and (1.0–0.062 mg/mL) for 3T3-L1 and L6 cell respectively for 60 min along with negative controls in glucose-free culture medium at 37 °C. At 10 min before the end of the treatment, 2-NBDG was added to a final concentration of 200 μg/mL in a glucose-free medium. At the end of treatment, the plate was centrifuged for 5 min at 400 × g at room temperature. The supernatant was aspirated and 200 μL of cell-based assay buffer was added to each well. The plate was centrifuged for 5 min at 400 × g at room temperature. The supernatant was aspirated and 100 μL of cell-based assay buffer was added to each well. The 2-NBDG taken up by cells can be detected with a fluorescent reading by (excitation/emission = 485/535 nm) by using Biotek Synergy H1 Multi-Mode Reader (Winooski, VT, USA). Three independent experimental values in duplicates are taken to determine the percentage of 2-NBDG uptake over controls.

#### Insulin secretion assay

Insulin release was determined using the monolayer of BRIN-BD11 clonal pancreatic cells. BRIN-BD11 cells were grown in RPMI1640 tissue culture medium containing 11.1 mmol glucose/l, 10%foetal calf serum and antibiotics (50,000 IU penicillin-streptomycin/l), and maintained at 37 °C in an atmosphere of 5% CO^2^ and 95% air. Then, 20 h prior to acute experiments, cells were harvested and seeded in 24-well plates at a density of 1 × 10^5^ cells/well. Following overnight attachment, the culture medium was removed and cells were preincubated for 40 min at 37 °C with 1 ml of Krebs ringer bicarbonate (KRB) buffer supplemented with 1.1 mM glucose and 1% bovine serum albumin. Samples were immediately done for subsequent insulin radioimmunoassay and were tested by using the Human Insulin Elisa Kit by Kono Biotech (Zhejiang, China).

### Molecular docking

#### Preparation of ligand

The set of ligand molecules studied in this work were obtained from the National Centre for Biotechnology Information (NCBI) PubChem database (http://PubChem.ncbi.nlm.nih.gov). The list of the ligands present in the extract that initially used are phlorizin (CID 6072), berberine (CID:2353), dimethylcaffeic acid (CID: 717531), monobenzone (CID: 7638), mundulone (CID:4587968), pomiferin (CID: 4871), scandenin (CID: 54676535), acarbose (CID: 41774), rosiglitazone (CID: 77999) and sitagliptin (CID: 4369359). These molecules were downloaded in Structure Data File (SDF) format and converted to Protein Data Bank (PDB) format.

#### Preparation of receptor

The crystal structure of α-glucosidase (PDB ID: 4J5T), DPP (IV) complex enzyme (PDB ID: 2P8S) and insulin receptor (IR) (PDB ID: 1IR3) was retrieved from Protein Data Bank (http://www.rcsb.org/pdb). These molecules were downloaded in Protein Data Bank (PDB) format and the water molecules, as well as other heteroatoms, were omitted from the complex protein molecule.

#### Docking

Molecular docking studies were carried out using AutoDock program to study the binding within the active site of each of the enzymes. The binding conformation was visualised using PyMOL. The grid size was set to 28 × 28 × 30 xyz points andgrid centre was designated at dimensions (x= −14.748, 16.602, 48.062, y= −27.504, 49.805, 51.582, z= −1.293, 21.889, 34.659, for 4J5T, 1HNY and 2P8S respectively. The grid centre was placed in the active site pocket centre. The grid boxes included the entire binding site of the enzyme and provided enough space for the ligand translational and rotational walk. A scoring grid is calculated from the ligand structure to minimise the computation time. After the complete execution of AutoDock, various conformations of the ligand in a complex with the receptor were obtained, which were finally ranked based on binding energy. The results of the interaction were analysed using a Protein-Ligand Interaction Profiler (PLIP) server (http://plip.biotec.tu-dresden.de/plip-web/plip/index). Results thus obtained were expressed in terms of free energy (kcal/mol) of protein-ligand binding.

### Statistical analysis

The data were expressed as mean ± SD values. Prism7 was used for statistical analysis. The cut off for significant variation was set at *p* < 0.05 using Duncan's test.

## Results

### α-Glucosidase inhibitory activity

**Figure 1.**
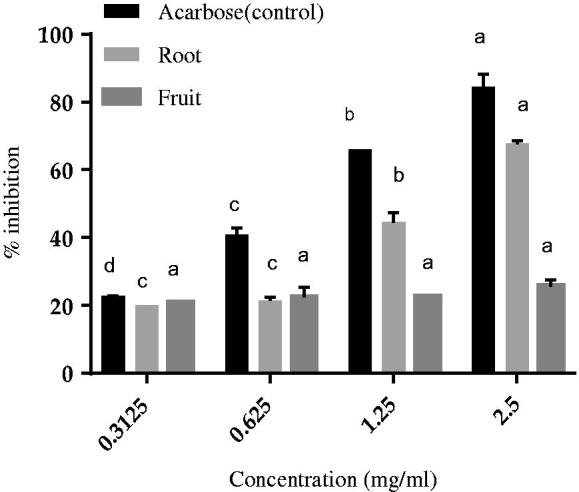
α-Glucosidase inhibitory effects of the extracts and the acarbose reference as a positive control. Values are expressed as the mean ± SD (*n* = 3). Different letters indicate significant differences (*p* < 0.05) within the same sample/control as increasing the concentration.

The percent of α-glucosidase inhibition activity of *C. latifolia* root and fruit extracts was plotted with the function of concentration in comparison with acarbose that acts as a positive control as shown in Figure 1, where the concentration of the sample is ranging from 0.3 to 2.5 mg/ml. The result indicates that root extracts have significantly more potential for inhibiting these enzymes which inhibit above 50% on the enzyme compared to the fruit extracts. The highest concentration of roots extract (2.5 mg/ml) inhibited the maximum percentage inhibition of α-glucosidase was nearly 56%. Meanwhile, fruit extracts have recorded the maximum inhibition activity with 22% at the maximum extract concentration (2.5 mg/ml) and for fruits extracts, it notified there was no significant difference as the concentration increased from 0.3125–0.625 mg/ml and after that, as increasing the concentration, there was significant difference reported. Even though fruit extracts give a much lower inhibition value as compared with root, it also showing good inhibition with the α-glucosidase enzyme. Acarbose appeared to be about 2-fold more active than root extract and 5 times more active that fruit extract. We also determined the half-maximum inhibitory concentration (IC_50_) for each fraction to quantify the inhibitory potential of the extracts, resulting in a 50% suppression of the original enzyme activity., where root extract was shown as a good inhibitor for a *α*-glucosidase enzyme with IC_50_ 2.72 mg/ml while the fruit extract recorded with IC_50_ 3.87 mg/ml.

### DPP (IV) inhibitory activity

**Figure 2.**
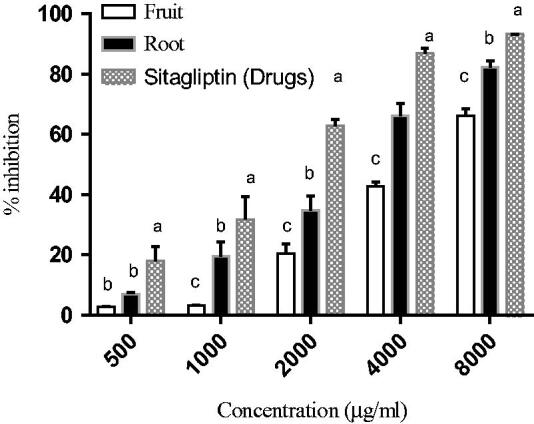
DPP (IV) inhibitory effects of the extracts and sitagliptin reference as a positive control. Values are expressed as the mean ± SD (*n* = 3). The different letter indicates there is a significant difference by comparing in each sample/drug under different concentrations (*p* < 0.05) according to ANOVA and Tukey’s tests.

The inhibitory potential of DPP (IV) by root and fruit of *C. latifolia* that also being compared with marketed synthetic drugs,sitagliptin which was used as a positive control in this assay was shown in Figure 2. The test concentration used was in the range from 250 to 3000 μg/ml. From the results, *C. latifolia* root extracts had recorded an inhibitory potential of DPP (IV) from 7.02 ± 0.52% to 66.15 ± 4.09% while fruit extract had inhibitory potential from 2.69 ± 0.25% to 42.79 ± 1.47%. DPP (IV) inhibitory potential of standard drugs, sitagliptin was found to be from 17.94 ± 4.73% to 86.96 ± 1.60%. The maximum inhibition that was achieved for root, fruits, and drugs at the highest concentration, 3 mg/ml were 66.15, 42.79, and 86.96% respectively. The results also showed that as increasing the extract’s concentration, the percentage of inhibition was also increased. There was a significant difference for the sample extracts and drugs, as were observed in all tested concentrations.

### Screening of active compound using LC-MS analysis

**Figure 3.**
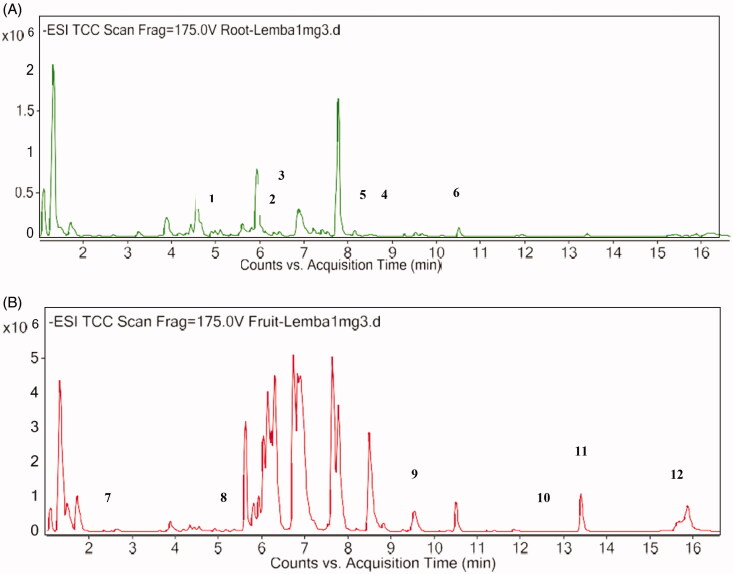
LC-MS chromatogram screening of *C. Latifolia.* (A) root extract and (B) fruit Extract. Peaks: 1, phlorizin; 2, scandenin; 3, mundulone; 4, hydroquinone; 5,dimethylcaffeic acid; 6, hordatine A; 7, berberine; 8, pomiferin; 9, hordatine A; 10, monobenzone; 11, frangulin B; 12, robustine.

A total 120 of compounds including known and unknown compounds were identified by LC-MS in the negative ion mode. Based on the results, peaks were labelled according to the order of their retention time, absorption wavelengths, *m*/*z* values, and tentative identification were aided by the existing literature.In particular, these compounds were phenolic (monobenzone) and identification as phenolic from benzene derivative (hydroquinone), flavonoid glycoside (phlorizin), prenylflavonoids (pomiferin), isoflavonoid (mundulone and scandenin) and in particular cinnamic acid derivative (dimethyl caffeic acid). The representative chromatograms in the screening of *C. latifolia* root and fruit extracts shown in Figure 3 and Table 1. In the analysis of root extract, the compound that was detected were as follows: phlorizin (*m/z* 477), scandenin (*m/z* 433), mundulone (*m/z* 433, 867), hydroquinone (*m/z* 109), dimethylcaffeic acid (*m/z* 683), hordatine A (*m/z* 532, 549, 565). Meanwhile, the compound that was detected for the fruit extract were as follows: berberine (*m/z* 1004), pomiferin (*m/z* 447), hordatine A (*m/z* 549), monobenzone (*m/z* 399), frangulin B (*m/z* 401), and robustine (*m/z* 183, 195, 207, 217, 221, 239).

**Table 1.** LC-MS analysis of *C. latifolia.*

Sample	Peak no.	Retention time (min)	Compound	Formula	Fragment ions
Root extract	1	5.185	Phlorizin	C21 H24 O10	*m/z* of 477
	2	6.589	Scandenin	C26 H26 O6	*m/z* of 433
	3	6.725	Mundulone	C26 H26 O6	*m/z* 433,867
	4	7.529	Hydroquinone	C6 H6 O2	*m/z* 109
	5	8.447	Dimethylcafeic acid	C11 H12 O4	*m/z* 683
Fruit extract	6	10.74	Hordatine A	C28 H38 N8 O4	*m/z* 532,549,565
	7	2.436	Berberine	C20 H17 NO	*m/z* 1004
	8	5.334	Pomiferin	C25 H24 O6	*m/z* 447
	9	9.732	Hordatine A	C28 H38 N8 O4	*m/z* 549
	10	12.962	Monobenzone	C13 H12 O2	*m/z* 399
	11	13.698	Frangulin B	C20 H18 O9	*m/z* 401
	12	15.95	Robustine	C12 H9 N O3	*m/z* 183,195,207, 217,221,239

### In vitro studies

#### Cell viability assessment in the presence of C. latifolia extract

The effect of root and fruit extracts on a mechanism in the antidiabetic pathway through glucose uptake assay and insulin secretion were investigate by MTT assay. The ability of the cells to survive a toxic insult has been the basis of the cell viability assessment. Cell viability assessment was undergone throughout varied extracts concentrations (0.25 mg/ml-2 mg/ml) that depends on the toxicity of the extracts in each of the cells. The comparison of IC_20_ and IC_50_ was summarised in Table 2. From the results, the toxicity for roots had recorded IC_20_ (153.21 ± 9.65 μg/ml) and IC_50_ (561.42 ± 6.22 μg/ml), a little bit higher than fruits extract with IC_20_ (295.67 ± 7.43 μg/ml) and IC_50_ (495.67 ± 11.31 μg/ml) in 3T3–L1 cell. With the treatment on L6 cells, roots had recorded IC_20_ (605.53 ± 12.33 μg/ml), IC_50_ (982.11 ± 5.53 μg/ml), while fruits extract with IC_20_ (625.54 ± 21.43 μg/ml), IC_50_ (893.41 ± 6.67 μg/ml). The results for in the cell viability in BRIN-BD11 cell was achieved with root extracts recorded IC_20_ (681.87 ± 15.42 μg/ml), IC_50_ (927.42 ± 5.81 μg/ml), while fruits extract with IC_20_ (649.82 ± 7.41 μg/ml), IC_50_ (1249.82 ± 15.34 μg/ml). Generally, the dose of each treatment in the extracts is partially based on the MTT assay where the safe concentration for the cell to be induced with tested extract and fractions. Thus, concentration up to 1000 μg/ml was employed in a further assay for *in vitro* studies.

**Table 2.** IC_20_ and IC_50_ values of *C. latifolia* root and fruit extract on 3T3-L1 preadipocytes, L6 muscle cell, and BRIN-BD11 pancreatic cell.

	3T3-L1		L6		BRIN-BD11	
Extracts	IC_20_ (μg/ml)	IC_50_ (μg/ml)	IC_20_ (μg/ml)	IC_50_ (μg/ml)	IC_20_ (μg/ml)	IC_50_ (μg/ml)
Root	153.21 ± 9.65	561.42 **±** 6.22	605.53 ± 12.33	982.11 **±** 5.53	681.87 ± 15.42	927.42 **±** 5.81
Fruit	295.67 **±** 7.43	495.67 **±** 11.31	625.54 ± 21.43	893.41 **±** 6.67	649.82 ± 7.41	1249.82 **±** 15.34

Data are expressed as mean ± standard deviation (*n* = 6).

#### Glucose uptake activity of C. latifolia extract in 3T3-L1 adipocyte and L6 muscle cell

**Figure 4.**
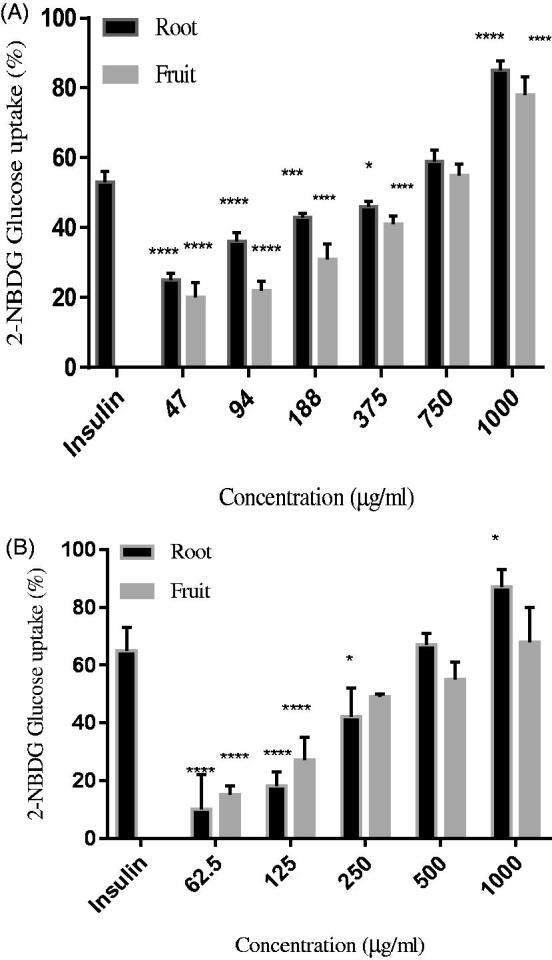
Percentage of glucose uptake after treatment with various concentration (A) 3T3-L1 adipocyte cell; (B) differentiated L6 muscle cell. Values are expressed as the mean ± SD (*n* = 6). *p < 0.05, **p < 0.01, ***p < 0.001 and ****p < 0.0001 compared to the control.

The result given by the uptake of glucose showed from the activity in 3T3–L1 and L6 cell line were shown in Figure 4. The result was compared with insulin (injectable antidiabetic) at a fixed concentration of 250 μg/ml. The given results show that the root extract achieved a higher percentage in both cell lines with 85 and 67% for L6 and 3T3-L1 cells respectively as compared with fruit extracts. In 3T3–L1 adipocyte cells, we found that the root extract of *C. latifolia* showed an increase of glucose uptake 750 and 1000 μg/mL while fruit extract recorded an increment of glucose uptake at concentration 1000 μg/ml when compared to the control insulin. However, based on results, we can observe that in order to get higher glucose uptake as same as insulin, the concentration of the extract was 4 fold higher than the concentration of insulin. The results revealed that there was a significant difference (*p* < 0.0001) increases in the uptake reaction were observed with the incubation with respective 47, 94, and 1000 µg/ml of root and 47, 94, 188, 375, and 1000 µg/ml. On the other hand, in L6 myoblast cells, we found that the root extract of *C. latifolia* showed an increase of glucose uptake 1000 μg/ml while fruit extract recorded a slight lower in glucose uptake at concentration 1000 μg/ml when compared to the control insulin. However, based on results, we can observe that in order to get higher glucose uptake as same as insulin, the concentration of the extract was 4-fold higher than the concentration of insulin that contributes to the above 80% uptake of glucose by L6 cell. The results revealed that there was a significant difference (*p* < 0.0001) increases in the uptake reaction were observed with the incubation with respective 62.5 and 125 µg/mL for both of the extracts.

#### Insulin secretion activity of C. latifolia extract in BRIN-BD11 pancreas cell line

**Figure 5.**
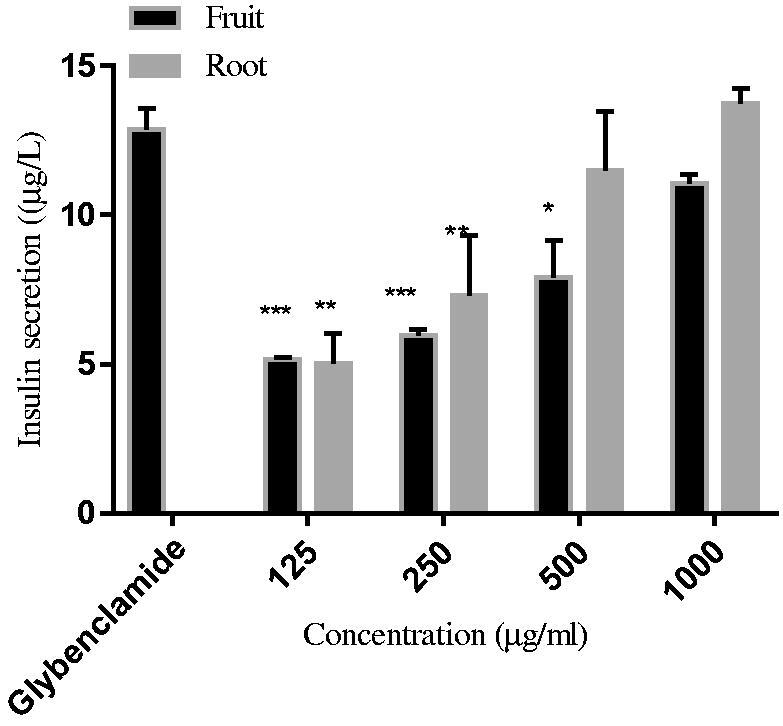
Insulin secretion assay for root and fruit of *C. latifolia* extracts. Values are expressed as the mean ± SD (*n* = 5). *p < 0.05, **p < 0.01, ***p < 0.001 and ****p < 0.0001 compared to the control.

The secretion of insulin assay was conducted by using the BRIN-BD11 cell line, which is a clonal insulin-secreting cell that responds to a variety of insulin-secreting pharmacological modulators. This cell line is mainly used to examine the *C. latifolia* extracts upon the insulin secretion mechanism assay. Insulin release by BRIN-BD11 cells was significantly increased in a dose-dependent manner for both extracts as the sample concentration (0.125–1 mg/ml) (Figure 5). In this mechanism of the assay, glibenclamide (oral antidiabetic drugs) acted as positive control. However, the concentration of glibenclamide was standardises at 0.5 mg/ml. Root extract subsequently increase with 5.02 ± 1.42 μg/ml, 7.32 ± 2.23 μg/ml, 11.48 ± 0.51 μg/ml, 13.74 ± 0.57 μg/ml at concentration 125, 250, 500, 1000 μg/ml, respectively. On the other hand, the secretion of insulin for fruit extract also subsequently increase with 5.16 ± 0.79, 5.96 ± 1.29, 7.91 ± 0.79, 11.06 ± 0.35 μg/ml at concentration 125, 250, 500, 1000 μg/ml, respectively. Even though root extract shows a slightly higher secretion of insulin as compared with glibenclamide that recorded with 12.88 ± 0.79 μg/ml at a concentration (500 μg/ml), but the extract concentration was 2-fold higher than glibenclamide. However, both of the extracts showed a positive result by having an increased insulin release that comparable with glibenclamide results.

### Molecular docking

**Figure 6.**
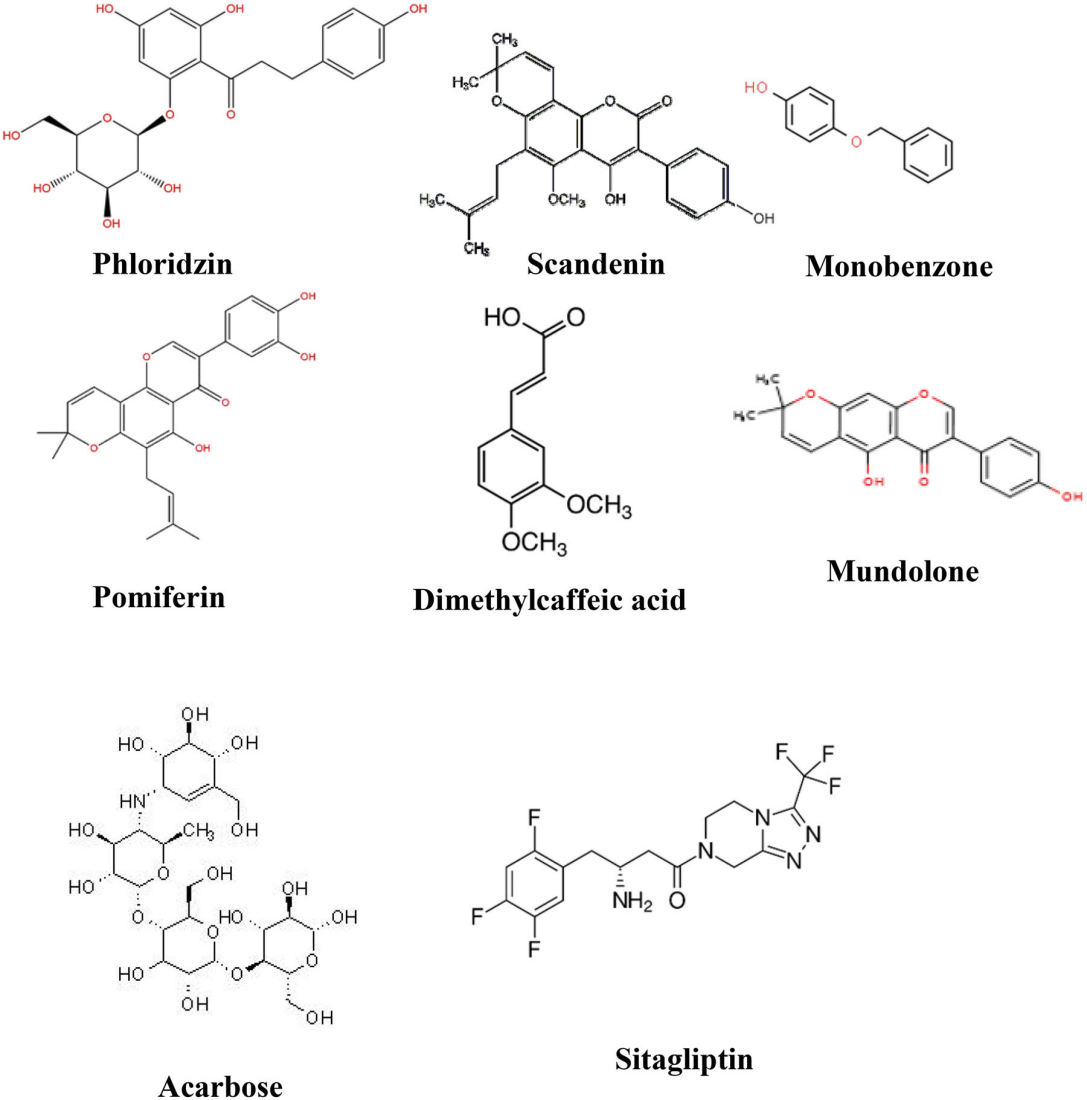
Compounds from SWE of *C. latifolia* with potential antidiabetic activity selected for *in silico* studies with selected anti-diabetic drugs.

**Figure 7.**
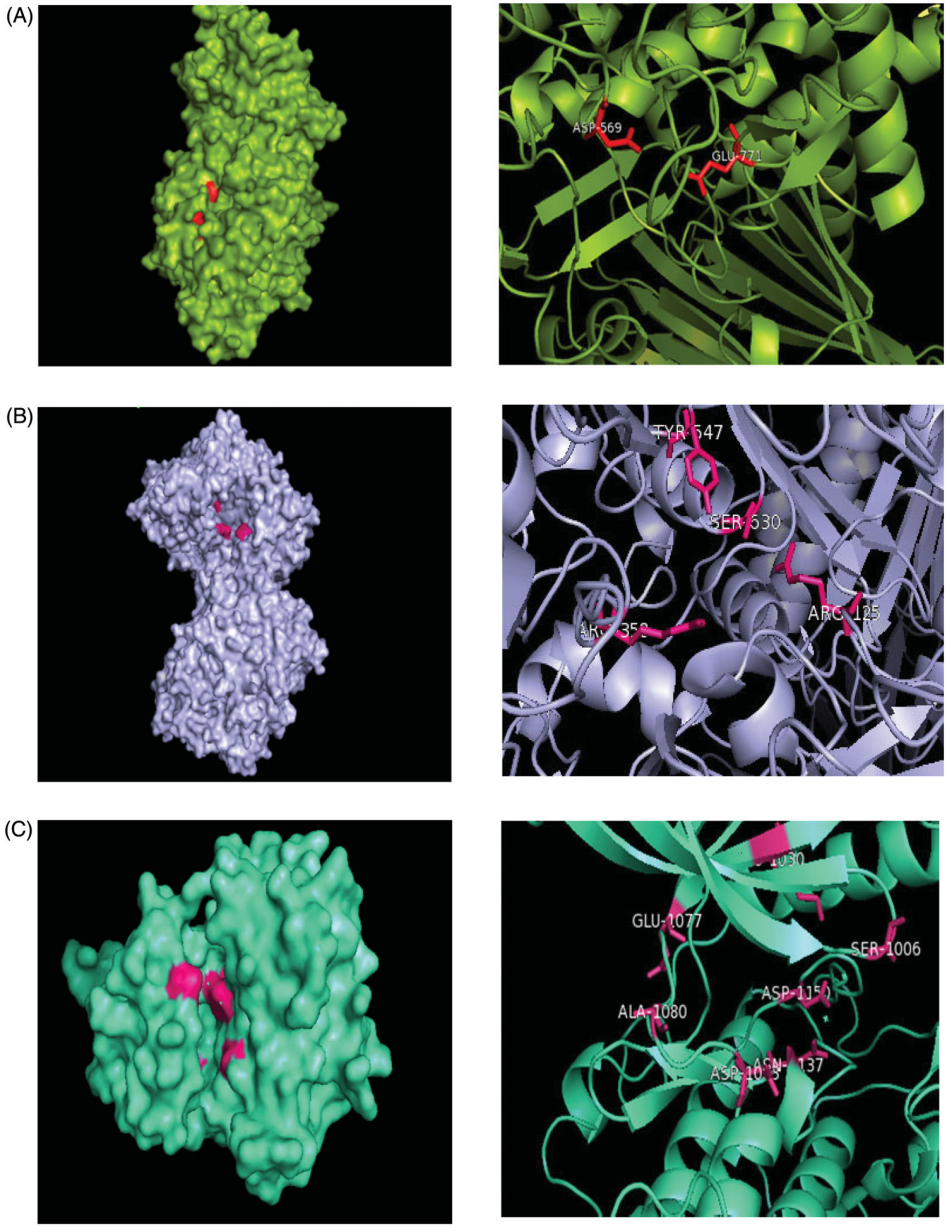
Active site in each targeted protein(A) Structure of α-glucosidase enzyme surface (green) with an active pocket site (red) and active site of the protein. (B) Structure of DPP (IV) enzyme surface (blue) with the active pocket site (pink) and active site of the protein.(C) Structure of Insulin receptor (green) with the active pocket site (pink) and active site of the protein.

**Figure 8.**
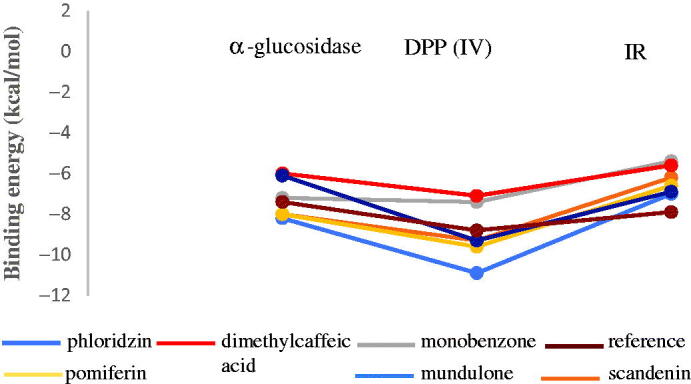
Binding energy between targeted protein and ligand. DPP (IV): Dipeptidyl Peptidase IV; IR: insulin receptor.

Molecular docking studies were used in order to investigate the binding confirmation to the selected compound. At the beginning of the process for docking, water molecules have been omitted in order to explore additional docking posses between compounds without the possible in the direct hydrogen bond formed. The molecular structure of selected ligands and active pocket sites for each of the receptors, 4J5T (α-glucosidase), 2P8S (DPP IV), and 1IR3 (insulin receptor, IR) were displayed in Figures 6 and 7, respectively. In order to explore binding energies (kcal/mol) and basis molecular interaction, all the ligands were docked with each of the proteins. The binding energy was indicative of the ligand's contribution and flexibility for the targeted protein. In terms of binding energy, the more negative the value of the standard free energy charge, the better the binding affinity of the ligand with the receptor, hence, the more stable the complex^20^. Therefore, the lowest binding energy scores represent the best protein-ligand binding stability compared to the highest energy score. The docking analysis with each of the receptor generate the binding energies on each ligands as follows: phlorizin (-8.2, −10.9, −7.0 kcal/mol), scandenin (-8.0, −9.3, −6.2 kcal/mol), pomiferin (-8.0, −9.6, −6.6 kcal/mol), berberine (-7.9, −8.9, -6.3 kcal/mol), monobenzone (-7.2, −7.4, −5.4 kcal/mol), mundulone (-6.1, −9.3, −6.9 kcal/mol), dimethycaffeic acid (-6.0, −7.1, −5.6 kcal/mol) for 4J5T, 2P8S and 1IR3, respectively. Meanwhile, the binding energies that were analysed for reference drugs acarbose (-7.4 kcal/mol), sitagtiptin (-8.8 kcal/mol), and insulin (-7.9 kcal/mol). The binding energies on each of the ligands were shown in Figure 8. The molecular interaction involved such as the number of hydrogen bond (H-bond), hydrophobic bonds, salt bridges, and π-staking as showed in Table 3. All details of the interaction including amino acid residue involved, bond length (Å), angle (^o^) and protein donor are given in appendices.

**Table 3.** Molecular docking analysis.

	H-bond	Hydrophobic bonds	Salt bridges	π-Staking
Enzyme 1 (4J5T)				
Compound 1 (acarbose)	9	1	–	–
Compound 2(phlorizin)	5	1	1	1
Compound 3 (pomiferin)	3	2		
Compound 4 (scandenin)	1	5	–	–
Compound 5 (mundulone)	1	2	–	–
Compound 6 (monobenzone)	–	–	–	3
Compound 7 (dimethylcaffeic acid)	–	2	1	1
Compound 8 (berberine)	–	–	1	1
Enzyme 2 (2P8S)				
Compound 1(phlorizin)	9	1	–	1
Compound 2 (mundulone)	3	1	–	–
Compound 3 (dimethylcaffeic acid)	2	1	1	1
Compound 4 (sitagliptin)	2	–	–	1
Compound 5 (pomiferin)	2	1	–	–
Compound 6 (berberine)	2	–	–	–
Compound 7(monobenzone)	1	–	–	1
Compound 8 (scandenin)	–	2		2
Enzyme 3 (1IR3)				
Compound 1(phlorizin)	6	–	–	–
Compound 2(mundulone)	4	1	–	–
Compound 3 (scandenin)	4	–	–	–
Compound 4 (pomiferin)	3	–	–	–
Compound 5(dimethylcaffeic acid)	3	–	–	1
Compound 6 (Insulin)	1	–	4	1
Compound 7 (berberine)	1	–	–	–
Compound 8(monobenzone)	–	–	–	1

**Figure 9.**
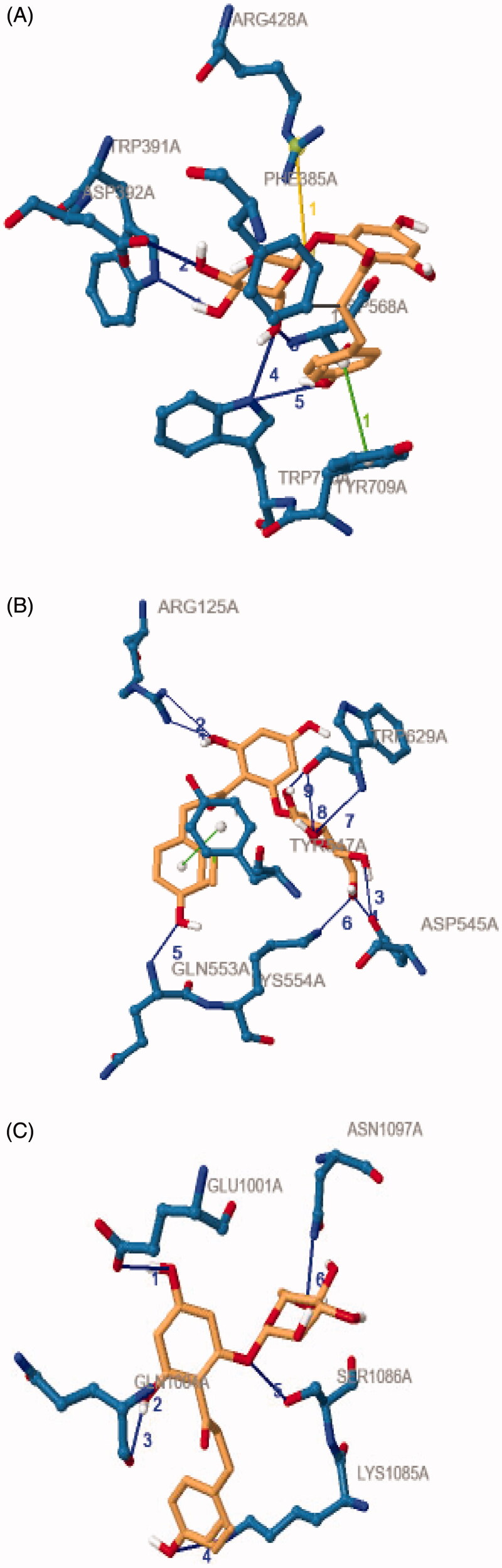
Best docked of complex molecule. (A) α-glucosidase (B) DPP (IV) and (C) IR. Blue structures indicate the enzyme/protein, the blue line indicates the hydrogen bonds formed between the residues, and the orange structures indicate the ligands involved respectively for both diagrams. The green line indicates the π-staking interactions and yellow lines indicate the formed salt bridge.

From the docking results with receptor 4J5T, among all the tested compounds, phlorizin was analysedH-bond interactions with Trp391, Asp392, Asp569 and Trp710, a hydrophobic interaction with Phe385, π-staking with Tyr709 and also salt bridges with Arg428. The amino acid residue of H-bond (Asp392 and Arg428), along with hydrophobic bonds, Leu563 were observed forming interaction with compound pomiferin. Scandenin and mundulone had a similar no of H-bond with Tyr709 and Arg428 respectively. Meanwhile, dimethylcaffeic acid, berberine and monobenzone have reported not forming any H-bond but dimethylcaffeic acid was supported with hydrophobic bonds (Asp568, Tyr709), a π-staking (Trp710) and a salt bridges (His561), berberine was supported with a π-staking (Tyr709) and a salt bridges (Asp568), and monobenzone was notified forming with π-staking interaction (Phe385, Phe389, Trp710). In the docking results with receptor 2P8S, phlorizin displayed H-bond interaction with Arg125, Asp545, Asp454, Gln553, Lys554, Trp629 amino acid residues and was stabilised with a hydrophobic bond (Tyr547) and π-staking (Tyr547). Pomiferin was notified forming H-bond (Asp556, Asp560), hydrophobic bond (Trp629) and mundulone were forming H-bond (Ser630, His740) along with hydrophobic bond (Trp629). Meanwhile, berberine and dimethylcaffeic acid formed H-bond with Gln553, Ser630 residues with a hydrophobic bond (Tyr547). Furthermore, monobenzone was forming a H-bond (Ser630) and a π-staking (Tyr547) while scandenin was reported with no H-bond formed but was supported with a hydrophobic bond (Trp629) along with Tyr547 residues for π-staking interaction. For docking analysis with 1IR3, phlorizin showed that formation of six H-bond (Glu1001, Gln1004, Lys1085, Ser1086, Asn1097), scandenin and mundulone with four H-bond (Arg1000, Arg1026, Ala1080) and (Ser1086, Asp1156, Thr1160) respectively. Besides, other compounds such as pomiferin and dimethylcaffeic acid formed three H-bond (Glu1047, Gly1152, Met1153) and (His1081, Asp1083, Ser1086) residues respectively. Berberine showed interaction with one H-bond (Gln1004) while monobenzone was analysed with none of H-bond formed but having a π-staking interaction (Tyr10). To sum up, we observe phlorizin forms the most stable binding in most of the receptor especially with DPP (IV) and was selected as a best-docked molecule as shown in Figure 9.

## Discussions

The coordinated function of several different tissues and organs depends on the optimal regulation of blood glucose. These include the gut, which is responsible for digestion and absorption of carbohydrates and pancreatic β-cells, which are responsible for the precise secretion of insulin. In the present study, the effect of antidiabetic activity were examined including the inhibitory potential of α-glucosidase, DPP (IV) enzyme, glucose uptake and secretion of insulin from the *C. latifolia* root and fruit extracts that were collected by using subcritical water extraction (SWE) technique. This research study also completed by a computational approach through molecular docking studies that reveal the binding interaction and other bonds that formed between the proteins with the receptor involved. Many earlier studies have revealed the role of plant extracts as a natural inhibitor that has a less adverse effect and is capable of reducing hyperglycaemia particularly in the inhibitory activity of the α-glucosidase enzyme^8^. From the results, the inhibitory activity on both of the extracts shows explicated a remarkable inhibition on the α-glucosidase enzyme by revealing the percentage inhibition by root extracts is closer with the percentage of inhibition by marketed anti-diabetic drugs, acarbose. Previously, the findings of the current study are in a good agreement with other research works indicate that SWE extracts are capable of influencing α-glucosidase inhibitory capacity^21^.

Besides, the presence of several phytochemicals such as flavonoids, phenolic with a greater number of hydroxyl groups that present in the compounds would have a significant inhibitory effect that boasts a greater inhibitory effect towards the α-glucosidase enzyme^22^. Significantly, the results on the inhibitory activity increased in conjunction with the increased of the concentration of the extract^23^. Previously, it has been reported that some studies shows a positive relationship between total polyphenol content and its ability to inhibit α-glucosidase^24^. Besides, the results given by using subcritical water extraction has a remarkable activity in inhibition of α-glucosidase. By applying hot extraction technique, the inhibition of α-glucosidase on *Meriandra bengalensis* has revealed that the inhibitory activity from water extracts achieved high inhibitory activity as compared with other organic solvents^24^. This can be related with the principal of water under subcritical state, where the polarity of water decrease as increasing the water temperature. This will causes the decrease of dielectric constant of water originally at ambient temperature is ε = 80 significantly reduced to (ε = 27 at 250 °C), which was pretty closed to the permittivity of ethanol (ε = 25) or methanol (ε = 33)^25^. Hence, these factors reducing the results of the gap produced between the solvent and SWE extraction methods. Therefore, the findings revealed that the extracts of *C. latifolia* from SWE extraction have potential in inhibiting α-glucosidase activity, which works as one of the natural inhibitors for α-glucosidase that associated with T2DM.

With insulin resistance being central in type 2 diabetes pathogenesis, a new alternative in the inhibition of DPP (IV) has proposed in order to stimulate the release of insulin. From the inhibitory activities of the extracts with DPP (IV), the results revealed that both of the extracts displayed a good inhibitory potential by having an increment in the percent of inhibition for the DPP (IV) enzyme.

Besides, it can be noticed that *C. latifolia* root extract acted as a better inhibitor as compared with the fruit extracts. This is possibly due to the inhibition from the bioactive compound, containing mixtures of several phenolics and flavonoids that contained in the root extracts that may acts as an inhibitor for the inhibition of the DPP (IV) enzyme^26^. In addition, some bioactive compounds containing in crude extracts can exert their properties in DPP (IV) inhibition because previous studies reported that subclasses in phenolic, flavonoid, terpenoids, and peptides were the most predominant compound as DPP (IV) inhibitors^27^. Whereby, the inhibition activity by the fruit extracts is probably due to the berberis compound that was present in the extract, where the activity of berberis is quite similar to DPP (IV) inhibitor, benzoquinolizines and it was demonstrated berberine significantly reduced the fasting blood glucose (FBG), lowered blood glucose level through increasing insulin receptor expression in T2DM^28^. The viability of the cell was assessed through MTT assay and the results were employed in determining the IC_20_ and IC_50_ of the cell growth. Fruit extracts proposed a lower toxicity effect at IC_50_ for 3T3–L1, L6, and BRIN-BD11 cell lines as compared with the root extract. Moreover, less toxicity of the sample was probably by applying the optimum condition of SWE that leads to high polarity and reduced toxicity. The sample’s concentration lower than 1 mg/ml was considered as a safe concentration to pursue *in vitro* assay activities in the glucose uptake and insulin secretion assays.

Medicinal plants enhance the glucose uptake by GLUT4 translocation and were proven by the *in vitro* glucose model. The L6 and 3T3-L1 cell lines are the best-characterized cellular model origin to study glucose uptake and GLUT4 translocation. The selection of cell line was based on the involved in the mechanism of action in the uptake of glucose where adipose tissue and skeletal muscle are now well established as a primary target site in glucose metabolism as well as regulation in whole-body glucose homeostasis and main targets for insulin-stimulated glucose uptake to control hyperglycaemia^29^. Skeletal muscle cell acts as a major primary site that was responsible for postprandial glucose uptake. In the skeletal muscle, insulin increases glucose uptake by increasing functional glucose transport molecules in the plasma membrane^30^. Furthermore, it is the most abundant tissue in the whole body and thus, the proper function of skeletal tissue is important to maintain normal blood glucose levels. In fact, L6 cells represent a good model for glucose uptake because they have been used extensively to elucidate the mechanisms of glucose uptake in muscle, have an intact insulin signalling pathway and express the insulin-sensitive GLUT4^31^. Based on our findings showed that root extract displays a better activity with the increased stimulation of the 2- NBDG uptake in both of the cell lines as compared with fruit extract. We can observe that the 2- NBDG uptake increased as the dose concentration of the sample increase. This is in agreement with previous studies, which have addressed the increment of 2- NBDG uptake in 3T3-L1 and L6 cell lines along with the increase in the sample treatments^32^. The presence of polyphenols including berberine, flavonoid glycoside (phlorizin), isoflavonoid (mundulone and scandenin) and cinnamic acid derivative (dimethyl caffeic acid) that containing in the extracts, contribute in promoting glucose absorption that can arise from their synergistic or complementary interactions with multiple targets in order to exercise their pharmacological behaviour. Firstly, berberine is shown to mediate its action by encouraging translocation of GLUT4 and increased activity of AMP-activated protein kinase (AMPK) in L6 myotubes and mediating anti-diabetic properties^33^. Besides, cinnamic acid derivatives precisely activate proteins phosphoinositide 3-kinase (P13K) pathway and exert its anti-diabetic effect by substantially enhance the uptake of glucose^34^. The finding from previous studies suggests that the introduction of a hydroxyl group on the benzene ring of cinnamic acid at the meta- and/or para-position increases the ability to stimulate glucose uptake in the cells^35^.

Apparently, the active compounds regulated in the extracts also contribute to promoting the effects by improving the secretion of insulin. Relatively, the observation made on the *in vitro* effect of the extracts on insulin secretion seen in BRIN-BD11 cell line, clearly establishes that root extracts appear to display a better activity in the secretion of insulin as compared with fruit extracts. The presence of phytochemicals in the extracts that facilitate the insulin release on pancreatic cell substantiates our observed effectiveness. With the presence of cinnamic acid derivatives, this active compounds capable in inducing Ca^2+^ in pancreatic β-cell and this indicates that the stimulating insulin secretion process by cinnamic acid is due to an increase in Ca^2+^ flow through the L-type Ca^2+^ channels without causing membrane depolarisation by closing the KATP channels^34^. The consequent depolarisation leading to the opening of Ca^2+^ channels and Ca^2+^ intracellular elevation mediating insulin secretory vesicle exocytosis^36^. These findings were in well correlate with other studies that reporting the enhancement in promoting insulin release that being stimulated with the presence of cinnamic acid^37^.

Consequently, these studies further confirmed with molecular docking, superimposition of the docked molecule to the receptor with α-glucosidase, DPP (IV) and insulin receptor (IR) crystal structure and related interactions. Lower binding energy is equal to better receptor binding of a ligand. Therefore, the lowest binding energy scores represent the best protein-ligand binding stability compared to the highest energy scores. It was revealed that phlorizin significantly has the lowest binding energy and the highest formation of H-bond in all the receptors involved. Practically, the conformation structure of the ligand with contains more hydroxyl groups due to the targeted protein happens to be very sensitive to the positions of hydroxyl and greatly leading to increase binding affinities with more tight binding and more potent inhibitory effects^38^. The activity of phlorizin was also supported from theprevious studies, which demonstrated the potential of phlorizin that binds near the active site pocket of the SGL2 protein, which meant the inhibitor could block the interaction between SGLT2 and glucose, and thus absorption and resulting in the normalisation of blood glucose^9^. In the α-glucosidase docking analysis, acarbose demonstrated the highest H-bond on the binding with the receptor. Indeed, acarbose is a well-known antidiabetic drug used to treat type 2 diabetes by inhibiting α-glucosidase, has been found to anchor α-glucosidase in the enzyme pocket (domain A) and to bind to residues like Phe384, Arg387, Trp391, Asp392, Arg428, Asp569, Tyr709, Glu771 resulting in a powerful inhibition of α-glucosidase. In the present study, we noticed that hydroxyl groups on sugar moiety of acarbose are favourable for the ligand to interact with the amino acid residues of the binding pocket. In addition, acarbose was observed to bind with the residues like Asp569 and Glu771 that was fixed at the active site of α-glucosidase. However, interesting findings were also be found with phlorizin that forms as the second-highest in the formation of H-bond followed by pomiferin, scandenin. mundulone that revealed the potential of phlorizin to bind at one of the pocket active sites of α-glucosidase, which at residue Asp569 and the binding of these bonds with the enzyme catalytic site play a major role that responsible for the inhibitory activity of α-glucosidase.

The binding at the active site plays a vital role in increasing the binding affinities between ligand-receptor. Phlorizin was found to form nine hydrogen bonds with DPP (IV) among the seven compounds, and its binding energy was the highest among the seven compounds. We observe that the binding of phlorizin forming two H- bonds with active site residues of DPP (IV), Arg125 contribute to the lowest binding energy formed due to the molecular shape and size of a compound that makes it easier to penetrate the binding pockets. Meanwhile, compounds such as monobenzone, mundulone, berberine and dimethylcaffeic acid were also observed with a quite high binding energy is probably due to the formation of H-bonds at the active site, residue like Ser630. We also notified that the majority of the compounds such as berberine, dimethylcaffeic acid, phlorizin, monobenzone, sandenin found to be exclusively involved in the π-staking interaction with one of DPP (IV) active site, Tyr547 as similar with sitagliptin. These factors claimed as one of reason on these compounds have the ability to mimic the binding interaction and found to follow the same interaction pattern as compared with sitagliptin. Theoretically, π-staking was analysed as the three top most binding interaction in protein-ligand binding including H-bonds, hydrophobic bonds and others that also enhance binding affinity^39^. It is believed that π-staking is considered as one of the natural keys in non-covalent interaction and the presence of these bonds is significant in many biological systems as solitary effects but also their interplay omnipresent^40^. Moreover, the docking with the insulin receptor (1IR3) was also included in these studies. Residues of amino acids, namely Ser1006, Lys1030, Asp1083, Met1079, Ala1080, and Glu1077, were the major residues of amino acids involved in internal ligand interaction and most of the active components in 1IR3. According to the molecular docking results, pomiferin and scandenin were the only compounds that were notified to interact with one of the active sites of 1IR3, which are Asp1083 and Ala1080 respectively through hydrogen bonding interaction. By interacting with the active site of IR, these compounds have the potential to act as IR activators therefore, adjust blood glucose to promote the utilisation of glucose. Besides, results also showed that for ligands with high-affinity scores were also influenced by the increase in the number of H-bond formed. The presence of H-bonds is definitely attributed to form a major interaction and regarded to be the main driving force of conformational change of the receptor upon ligand binding. To the best of our knowledge, our present studies were found to be as the first studies that revealed the molecular docking with the compounds that present in *C. latifolia* extracts and this works is based on continuation from our previous studies that involving with antioxidant activity and screening of compounds by LC-MS analysis^16^. Thus, these studies have been pointed out the potential of phlorizin by inhibiting the activity of α-glucosidase and DPP (IV) enzyme as the new alternative for antidiabetic drugs that potentially being used in the treatment for type 2 diabetes.

## Conclusion

As a conclusion remarks, the results from our current study showed that the root and fruit extracts of *C. latifolia* likely to exert its anti-diabetic properties through inhibitory activity by α-glucosidase that involves in the digestion of carbohydrate mechanism and DPP (IV) enzyme in intestine that stimulating the secretion of insulin and reducing glucose level in the bloodstream. Additionally, both extracts were also observed to enhance in stimulating glucose uptake and insulin secretion. Briefly, the results also highlighted root extracts producing better antidiabetic activities as compare with fruit extracts. The effects of phytochemical compounds that enhance in antidiabetic activity were also being studied thoroughly by molecular docking that revealed the interesting findings of phlorizin compounds as the promising candidates which dock well with targeted protein and responsible in inhibitory activities. In fact, the quantification of each of the compound from the results on the screening from LCMS analysis was still in the progress. However, future studies should address the molecular mechanisms that may be related to the metabolic pathway for treating diabetes and isolation of the active constituents will be interesting to explore and can be considered for developing into a potent antidiabetic drug.

## Data Availability

The data used to support the findings of this study are available from the corresponding author upon request.

## References

[1] Okur ME, Karantas ID, Siafaka PI. Diabetes mellitus: a review on pathophysiology, current status of oral pathophysiology, current status of oral medications and future perspectives. ACTA Pharmaceutica Sciencia 2017;55:61.

[2] Cho N, Shaw JE, Karuranga S, et al. IDF diabetes atlas: global estimates of diabetes prevalence for 2017 and projections for 2045. Diabet Res Clin Pract 2018;138:271–81. 10.1016/j.diabres.2018.02.023 29496507

[3] Yuen L, Saeedi P, Riaz M, et al. Projections of the prevalence of hyperglycaemia in pregnancy in 2019 and beyond: Results from the International Diabetes Federation Diabetes Atlas. Diabetes Res. Clin. Pract 2019;157:107841. 10.1016/j.diabres.2019.107841 31518656

[4] Olokoba AB, Obateru OA, Olokoba LB. Type 2 diabetes mellitus: a review of current trends. Oman Med J 2012;27:269–73. 23071876 10.5001/omj.2012.68 PMC3464757

[5] Min SH, Yoon JH, Moon SJ, et al. Combination of sodium-glucose cotransporter 2 inhibitor and dipeptidyl peptidase-4 inhibitor in type 2 diabetes: a systematic review with meta-analysis. Sci Rep 2018;8:4466. 29535389 10.1038/s41598-018-22658-2 PMC5849757

[6] Wang Q, Long M, Qu H, et al. DPP-4 inhibitors as treatments for type 1 diabetes mellitus: a systematic review and meta-analysis. J Diabet Res 2018;2018:5308582. 10.1155/2018/5308582 PMC5817360 29507862

[7] Oboh G, Isaac AT, Akinyemi AJ, Ajani RA. Inhibition of key enzymes linked to type 2 diabetes and sodium nitroprusside induced lipid peroxidation in rats’ pancreas by phenolic extracts of avocado pear leaves and fruit. Int J Biomed Sci 2014;10:208–16. 25324703 PMC4199475

[8] Bhatia A, Singh B, Arora R, Arora S. *In vitro* evaluation of the α-glucosidase inhibitory potential of methanolic extracts of traditionally used antidiabetic plants. BMC Complement Alt Med 2019;19:74. 10.1186/s12906-019-2482-z PMC6434821 30909900

[9] Wang F, Zhang Y, Yu T, et al. Oat globulin peptides regulate antidiabetic drug targets and glucose transporters in Caco-2 cells. J Funct Foods 2018;42:12–20.

[10] Gajbhiye RL, Ganapathy A, Jaisankar P. A review of Α-glucosidase and Α-amylase inhibitors for type 2 diabetes isolated from some important indian medicinal plants. Ann Clin Pharmacol Ther 2018;1:1003.

[11] Jiang M, Yan H, He R, Ma Y. Purification and a molecular docking study of α-glucosidase-inhibitory peptides from a soybean protein hydrolysate with ultrasonic pretreatment. Eur Food Res Technol 2018;244:1995–2005.

[12] Natarajan A, Sugumar S, Bitragunta S, Balasubramanyan N. Molecular docking studies of (4 Z, 12 Z)-cyclopentadeca-4, 12-dienone from Grewia hirsuta with some targets related to type 2 diabetes. BMC Complement Alt Med 2015;15:73. 10.1186/s12906-015-0588-5 PMC4378231 25885803

[13] Nair AS, Kavrekar V, Mishra A. *In vitro* studies on alpha amylase and alpha glucosidase inhibitory activities of selected plant extracts. Eur J Exp Biol 2013;3:128–32.

[14] Ishak NA, Ismail M, Hamid M, et al. Antidiabetic and hypolipidemic activities of curculigo latifolia fruit: root extract in high fat fed diet and low dose STZ induced diabetic rats. Evid Based Complement Alt Med 2013;2013:1–12. 10.1155/2013/601838 PMC3671281 23762147

[15] Ooi DJ, Chan KW, Ismail N, et al. Polyphenol-rich ethyl acetate fraction of Molineria latifolia rhizome restores oxidant-antioxidant balance by possible engagement of KEAP1-NRF2 and PKC/NF-κB signalling pathways. J Funct Food 2018;42:111–21.

[16] Zabidi NA, Ishak NA, Hamid M, Efliza Ashari S. Subcritical water extraction of antioxidants from *Curculigo latifolia* root. J Chem 2019;2019:1–10.

[17] Kazeem MI, Ogunbiyi JV, Ashafa AO. *In vitro* studies on the inhibition of α-amylase and α-glucosidase by leaf extracts of *Picralima nitida* (Stapf). Trop J Pharm Res 2013;12:719–25.

[18] Foo JB, Yazan LS, Tor YS, et al. Induction of cell cycle arrest and apoptosis in caspase-3 deficient MCF-7 cells by *Dillenia suffruticosa* root extract via multiple signalling pathways. BMC Complement Alt Med 2014;14:197 10.1186/1472-6882-14-197 PMC4096536 24947113

[19] Jung CH, Lee DH, Ahn J, et al. γ-Oryzanol enhances adipocyte differentiation and glucose uptake. Nutrients 2015;7:4851–61. 26083118 10.3390/nu7064851 PMC4488819

[20] Iman M, Saadabadi A, Davood A. Molecular docking analysis and molecular dynamics simulation study of ameltolide analogous as a sodium channel blocker. Turk J Chem 2015;39:306–16.

[21] Sallau AB, Yakubu R ,Aliyu SM ,Salihu A. *In vitro* effect of terpenoids-rich extract of *Momordica charantia* on alpha glucosidase activity. Vitae 2018;25(3):148–153.

[22] Yusro F, Ohtani K, Kubota S. Inhibition of α-glucosidase by methanol extracts from wood bark of Anacardiaceae, Fabaceae, Malvaceae and Phyllanthaceae plants family in West Kalimantan, Indonesia. Kuroshio Sci 2016;9(2):108–22.

[23] Alam MA, Zaidul ISM, Ghafoor K, et al. *In vitro* antioxidant and, α-glucosidase inhibitory activities and comprehensive metabolite profiling of methanol extract and its fractions from *Clinacanthus nutans*. BMC Complement Alt Med 2017;17:181. 10.1186/s12906-017-1684-5 PMC5374668 28359331

[24] Kidane Y, Bokrezion T, Mebrahtu J, et al. *In vitro* inhibition of-amylase and-glucosidase by extracts from *Psiadia punctulata* and *Meriandra bengalensis*. Evid Based Complement Alt Med 2018;2018:2164345. 10.1155/2018/2164345 PMC6077584 30108648

[25] Shah MA, Jakkawanpitak C, Sermwittayawong D, Panichayupakaranant P. Rhinacanthins-rich extract enhances glucose uptake and inhibits adipogenesis in 3T3-L1 adipocytes and L6 myotubes. Pharmacog Mag 2017;13:S817. 10.4103/pm.pm_236_17 PMC5822505 29491638

[26] Fathima HM, Thangavelu L, Roy A. Anti-diabetic activity of cassia fistula (alpha amylase–inhibitory effect). J Adv Pharm Educ Res 2018;8:13.

[27] Lin SR, Chang CH, Tsai MJ, et al. The perceptions of natural compounds against dipeptidyl peptidase 4 in diabetes: from *in silico* to *in vivo*. Ther Adv Chronic Dis 2019;10:2040622319875305. 31555430 10.1177/2040622319875305 PMC6753520

[28] Chakrabarti S, Gilles RP, Lazarova E. Partial cooperation in strategic multi-sided decision situations. Theory Dec 2018;85:455–78.

[29] Nastić N, Švarc-Gajić J, Delerue-Matos C, et al. Subcritical water extraction as an environmentally-friendly technique to recover bioactive compounds from traditional Serbian medicinal plants. Indust Crops Product 2018;111:579–89.

[30] Das SV, Mooventhan A, Manjunath NK. A study on immediate effect of cold abdominal pack on blood glucose level and cardiovascular functions in patients with type 2 diabetes mellitus. J Clin Diag Res 2018;12(3).

[31] Arha D, Ramakrishna E, Gupta AP, et al. Isoalantolactone derivative promotes glucose utilization in skeletal muscle cells and increases energy expenditure in db/db mice via activating AMPK-dependent signaling. Mol Cell Endocrinol 2018;460:134–51. 28736255 10.1016/j.mce.2017.07.015

[32] Telapolu S, Kalachavedu M, Punnoose AM, Bilikere D. MD-1, a poly herbal formulation indicated in diabetes mellitus ameliorates glucose uptake and inhibits adipogenesis–an *in vitro* study. BMC Complement Alt Med 2018;18:113. 10.1186/s12906-018-2177-x PMC5879540 29606113

[33] Prasatha GS, Rahinia S, Srinivasana PT, Subramanianb S. *In vitro* antioxidant and glucose uptake potential of *Tinospora cordifolia* leaves extract. Der Pharmacia Lettre 2016;8:132–9.

[34] Adisakwattana S. Cinnamic acid and its derivatives: mechanisms for prevention and management of diabetes and its complications. Nutrients 2017;9:163. 10.3390/nu9020163 PMC5331594 28230764

[35] Chang HK, Hsu FL, Liu IM, Cheng JT. Stimulatory effect of cinnamic acid analogues on alpha1A-adrenoceptors *in-vitro*. J Pharm Pharmacol 2003;55:833–7. 12841945 10.1211/002235703765951456

[36] Bharucha B, Dwivedi M, Laddha NC, et al. Antioxidant rich flavonoids from Oreocnide integrifolia enhance glucose uptake and insulin secretion and protects pancreatic β-cells from streptozotocin insult. BMC Complement Alt Med 2011;11:126. 10.1186/1472-6882-11-126 PMC3267669 22169757

[37] Hafizur RM, Hameed A, Shukrana M, et al. Cinnamic acid exerts anti-diabetic activity by improving glucose tolerance *in vivo* and by stimulating insulin secretion *in vitro*. Phytomedicine 2015;22:297–300. 25765836 10.1016/j.phymed.2015.01.003

[38] Perilla JR, Goh BC, Cassidy CK, et al. Molecular dynamics simulations of large macromolecular complexes. Curr Opin Struct Biol 2015;31:64–74. 25845770 10.1016/j.sbi.2015.03.007 PMC4476923

[39] Bueschbell B, Barreto CA, Preto AJ, et al. A complete assessment of dopamine receptor-ligand interactions through computational methods. Molecules 2019;24:1196. 10.3390/molecules24071196 PMC6479630 30934701

[40] Frontera A, Quiñonero D, Deyà PM. Cation–π and anion–π interactions. Wiley Interdiscip Rev Comput Mol Sci 2011;1:440–59.

